# Leveraging a chromosomal instability-based signature to predict the prognosis and immune landscape of breast cancer

**DOI:** 10.1016/j.gendis.2025.101924

**Published:** 2025-11-06

**Authors:** Huiling Wang, Huijuan Dai, Yaohui Wang, Qiong Wu, Mingxi Zhu, Wenjin Yin, Jinsong Lu

**Affiliations:** Department of Breast Surgery, Renji Hospital, School of Medicine, Shanghai Jiao Tong University, Shanghai 200127, China

**Keywords:** Breast cancer, Chromosomal instability, Prognosis, Prognostic signature, Tumor immune microenvironment

## Abstract

Chromosomal instability (CIN) significantly impacts the tumor progression and tumor immune microenvironment (TIME). However, few researchers have focused on CIN variables in predicting prognosis and the immune landscape of breast cancer. Through unsupervised consensus clustering, the TCGA-BRCA cohort was categorized into two clusters based on the CIN25 gene signature. After identifying the two clusters’ differentially expressed genes, we sequentially performed univariate Cox, LASSO, and multivariate Cox regression analyses to construct a 13-gene signature, termed “CIN score”. Then, the breast cancer patients were divided into low- and high-CIN score groups. The differences in survival outcome, clinicopathological parameters, TIME, and drug sensitivity between the two groups were further investigated. The high-CIN score group had unfavorable clinicopathological features and overall survival. TIME analysis indicated that the low-CIN score group had increased expression of immune checkpoint genes and infiltration of immune cells, suggesting that immunotherapy was more likely to benefit the low-CIN score group. Additionally, drug sensitivity analysis indicated the high-CIN score group has lower sensitivity to several commonly used chemotherapeutic, endocrine, and targeted agents than the low-CIN score group. The novel gene signature, CIN score, identified in our research, offers a novel tool to predict the prognosis, TIME, and drug responsiveness in breast cancer, thus providing insights into immunotherapy decision-making and contributing to the precision treatment in breast cancer.

## Introduction

Breast cancer (BC) is a highly heterogeneous disease that affects women all over the world. Globally, BC is still the most frequently diagnosed cancer in women, accounting for almost 30% of female malignancies.^1^^,^^2^ Various treatment approaches, such as chemotherapy, targeted therapy, surgery, radiation, and endocrine therapy, are available for BC.^3^, ^4^, ^5^ Despite significant treatment advances, a considerable number of BC patients eventually experience *de novo* or acquired resistance to treatment, resulting in disease progression or distant metastasis.^6^^,^^7^ Tumor progression presents a nearly insurmountable obstacle to long-term cancer management. Those with metastatic BC usually have a dismal prognosis.

Chromosomal instability (CIN) is considered to be a hallmark of cancer. It is defined as chromosome segregation errors that persist over successive cell divisions.^8^ Approximately 90% of solid tumors are estimated to exhibit some degree of aneuploidy.^9^ The genomes of BC are frequently tetraploid or near-triploid. In addition, segmental copy number alterations, such as *ERBB2* amplification, are common in BC genomes.^10^^,^^11^ Researchers classified CIN into numerical chromosomal instability (nCIN) and structural chromosomal instability (sCIN).^12^^,^^13^ nCIN, often referred to as aneuploidy, is the gain or loss of entire chromosomes.^9^^,^^14^ sCIN refers to chromosomal rearrangements or the gain or loss of chromosome fragments. CIN can be caused by defective mitosis, transcriptional stress, oncogene-induced replication stress, and DNA damage repair defects.^15^, ^16^, ^17^ Due to the difficulty of evaluating CIN, Carter et al proposed the CIN25 gene signature for the CIN assessment in 2006.^18^ The authors showed that CIN25 could reflect the level of CIN and predict the prognosis of BC. With the development of bioinformatics in recent years, we recognize that thoroughly elucidating how CIN impacts prognosis and tumor immune microenvironment (TIME) of BC using new bioinformatics methods would inform us of a new clue to target CIN in BC therapy. However, few researchers have focused on utilizing CIN-related variables to predict BC’s prognosis and immune landscape. Therefore, we aimed to exploit new bioinformatics methods to comprehensively delineate the CIN25 signature in BC and construct a simplified CIN-related risk model.

Immunotherapy, specifically targeting the TIME rather than the tumor cells, has shown promise in BC treatment.^19^ Immune checkpoint inhibitors (ICIs), such as PD-1 inhibitor pembrolizumab, have been tested in triple-negative breast cancer (TNBC) clinical trials.^20^ The event-free survival of early TNBC is improved by pembrolizumab plus neoadjuvant chemotherapy followed by adjuvant pembrolizumab, which is independent of tumor PD-L1 expression.^21^ For PD-L1^+^ metastatic TNBC, a new standard of care for the first-line treatment is pembrolizumab plus chemotherapy, which improves overall survival (OS).^22^ Because BC is a highly heterogeneous disease, it is still challenging to identify patients who will benefit from ICIs.^23^ The response to ICIs may be higher in immune-enriched tumors, characterized by high tumor-infiltrating lymphocytes and immune checkpoint expression. Previous reports have revealed that aneuploidy is associated with a poor response to ICIs in melanoma patients.^24^ This provides a rationale for exploring more effective biomarkers based on CIN variables to identify BC patients most likely to respond favorably to ICIs.

**Figure 1:**
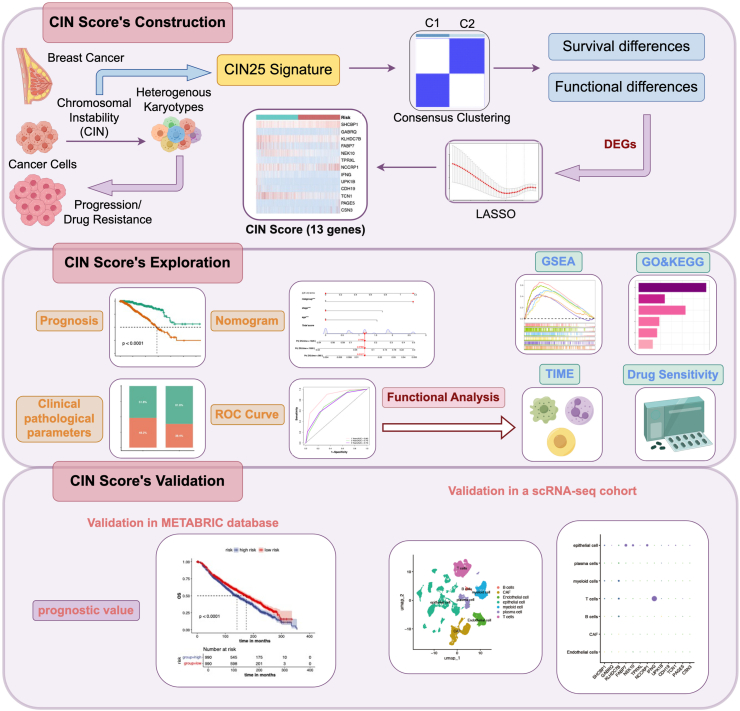
Flow chart of this study.

In this study, we performed RNA transcriptome analysis in The Cancer Genome Atlas (TCGA) database and proposed a novel CIN-related gene signature, CIN score. Various algorithms, including machine learning, enrichment analysis, and immune infiltration, were applied to evaluate the ability of the CIN score to predict BC survival outcomes and the immune landscape. These results were validated by an external cohort, the Molecular Taxonomy of Breast Cancer International Consortium (METABRIC). Moreover, we explored the expression patterns of the CIN score in different cell types of BC through single-cell sequencing data (scRNA-seq). The workflow of this study is shown in Figure 1.

## Materials and methods

### Data gathering

Transcriptomic data and clinical profiles of 1091 BC samples and 113 normal samples were downloaded from The Cancer Genome Atlas (TCGA) database (https://www.cancer.gov/ccg/research/genome-sequencing/tcga). RNA-seq data and clinical information were extracted from the METABRIC and GEO databases for validation. In addition, to explore the expression patterns of the genes in the CIN score in different cellular subtypes, scRNA-seq data (GSE176078) of 26 primary BC samples were acquired from the Gene Expression Omnibus (GEO) database (https://www.ncbi.nlm.nih.gov/geo).

The CIN25 gene signature includes the following genes: TRIP13, NCAPD2, ESPL1, CDK1, MELK, PRC1, KIF20A, TOP2A, TTK, TPX2, UBE2C, MCM7, MCM2, RFC4, FEN1, CDC45, FOXM1, RAD51AP1, H2AFZ, MAD2L1, PCNA, RNASEH2A, TGIF2, CCT5, and CCNB2.^18^

### Mutation landscape and copy number variant analysis of the CIN25 gene signature

Somatic mutation profiles and copy number variation data were gathered from the UCSC Xena database (https://xena.ucsc.edu/). We plotted a waterfall diagram using the “maftools” package to show the mutation landscape of the CIN25 gene signature in BC patients. The copy number variation frequency of the CIN25 gene signature was analyzed through GISTIC_2.0 and then visualized.

### Unsupervised consensus clustering

Unsupervised clustering was performed via the “ConsensusClusterPlus” package based on the expression of the CIN25 gene signature.^25^ The “DESeq2” package was then utilized to analyze differentially expressed genes (DEGs) between the two clusters.^26^ Significant differences were defined as *P*-value < 0.05 and log fold change (|log fold-change|) > 1.

### Construction of the CIN score

To obtain the prognostic DEGs, we first performed a univariate Cox analysis. The LASSO algorithm was performed via the “glmnet” R package,^27^ and then a multivariate Cox analysis was performed to derive the independent predictive genes. A final set of 13 genes (SHCBP1, GABRQ, KLHDC7B, FABP7, NEK10, TPRXL, NCCRP1, IFNG, UPK1B, CDH19, TCN1, PAGE5, CSN3) was included to construct a prognostic gene signature, termed “CIN score”. CIN score = $\sum_{i=1}^{n}{\text{Coe}}_{i}*{\text{Exp}}_{i}$, where Coei refers to the coefficients of the genes, and Expi refers to the mRNA expression of genes.

For subsequent analyses, BC patients were separated into low- and high-CIN score groups based on the median CIN score value.

### Establishment of the CIN-related nomogram

CIN score and other clinicopathological factors were incorporated into univariate and multivariate Cox regression analysis to identify independent prognostic factors. A nomogram predicting the 1-, 3-, and 5-year OS was established based on the independent predictors. The nomogram plot was shown by the “rms” R package. Calibration curves, time-dependent receiver operating characteristic (ROC) curves,^28^ and decision curve analysis were used to validate the nomogram’s prediction accuracy.

### Functional enrichment analysis

Functional enrichment analyses were performed via the “DAVID” website (https://david.ncifcrf.gov/), focusing on GO and KEGG pathways. Additionally, gene set enrichment analysis (GSEA)^29^ and gene set variation analysis (GSVA)^30^ were performed to estimate the signaling pathway activity. All pathways were obtained from the MSigDB (Molecular Signatures Database).

### Tumor microenvironment analysis

The ESTIMATE algorithm calculated the immune score and stromal score.^31^ Additionally, the Tumor Immune Estimation Resource (TIMER), quantification of the Tumor Immune contexture from RNA-seq data (quanTIseq), cell-type identification by estimating relative subsets of RNA transcripts (CIBERSORT), and single-sample gene set enrichment analysis (ssGSEA) methods were used to calculate the immune cell abundance via the “IOBR” R packages.^32^ Immunophenoscore (IPS) is the four gene categories that reflect tumor immunogenicity.^33^ Using IPS data from The Cancer Immunome Atlas (TCIA) (https://tcia.at/home), we compared the difference in potential immunotherapy response between the low- and high-CIN score groups.

### Prediction of drug sensitivity

The “oncoPredict” R package calculated the half-maximal inhibitory concentration (IC50) values for the commonly used chemotherapeutic, endocrine, and targeted agents.^34^ The drug sensitivity was compared between the low- and high-CIN score groups.

### Single-cell RNA sequencing analysis

To validate whether the marker genes could discriminate different cell types in BRCA, we downloaded scRNA-seq data (GSE176078) from the GEO database. To convert 10 × scRNA-seq data as a Seurat object and carry out quality control of the raw counts, the “Seurat” R package^35^ was adopted. Then, the “FindVariableFeatures” function was used to filter the top 2000 highly variable genes. Uniform manifold approximation and projection (UMAP)^36^ was used to visualize each cell type. The CellChat package was employed to infer the communication network between epithelial cells and stromal cells.

### Statistical analyses

All data analyses were performed using R software (version 4.3.2). Comparisons between the two groups were performed using a two-tailed Wilcoxon test if not specifically stated. Survival differences were visualized by the Kaplan–Meier method and assessed using the log-rank test. A *P*-value < 0.05 was considered statistically significant.

## Results

### The genetic characteristics and transcriptomic landscape of the CIN25 signature

**Figure 2:**
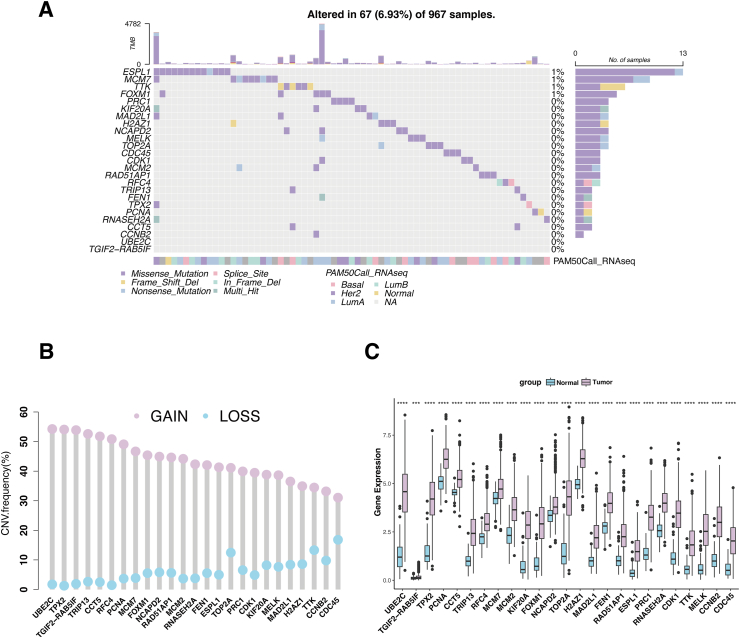
The genetic characteristics and transcriptomic landscape of the CIN25 signature. **(A)** The mutation landscape of the CIN25 signature in the TCGA-BRCA database. Different colors in the OncoPlot stand for different mutation types. The lower label indicates the molecular subtype of each patient. **(B)** The copy number variation (CNV) frequency of the CIN25 signature. **(C)** The mRNA expression of the CIN25 signature in BC tumors and normal breast tissues. ∗*P* < 0.05, ∗∗*P* < 0.01, ∗∗∗*P* < 0.001, and ∗∗∗∗*P* < 0.0001.

The CIN25 signature proposed by Carter et al includes 25 genes correlated with CIN. However, the genetic characteristics and transcriptomic landscape of the CIN25 signature in BC have not been delineated yet. In the genetic variation analysis, somatic mutations were found in 67 (6.93%) of the 967 samples, with missense mutations being the most frequent (Fig. 2A). Then, we analyzed the copy number variation frequency in the CIN25 signature and found that amplification of the copy number was more frequent than the loss of the copy number (Fig. 2B). Compared with the normal samples, the expression of the CIN25-related genes in tumor samples was up-regulated (*P* < 0.05; Fig. 2C).

### Unsupervised consensus clustering analysis based on CIN25 signature

**Figure 3:**
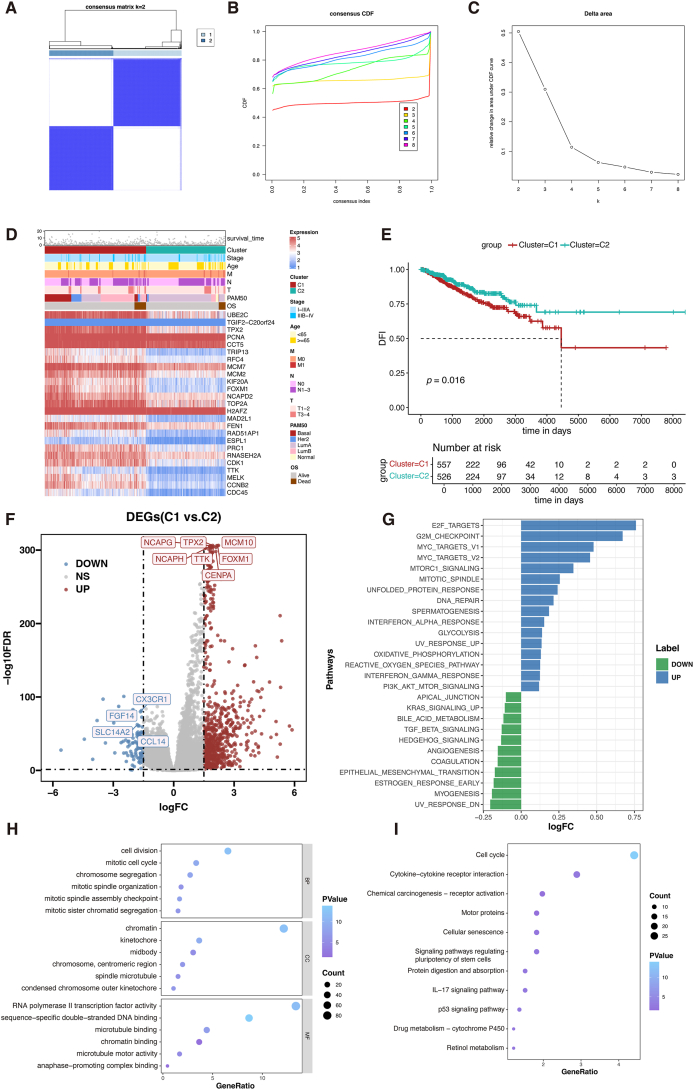
Unsupervised clustering of CIN25 gene signature in BC patients. **(A)** The TCGA-BRCA cohort was divided into two clusters when *k* = 2, based on the CIN25 gene signature. **(B)** Cumulative distribution function (CDF) plots illustrate consensus distributions for each k value. **(C)** Delta area curve of consensus clustering. **(D)** Heatmap of CIN25 gene expression in different clusters. **(E)** The disease-free interval (DFI) Kaplan–Meier curve of different clusters. **(F)** Volcano plot of differentially expressed genes (DEGs) between the two clusters, with cluster 2 as control (blue: down-regulated DEGs; red: up-regulated DEGs; grey: unchanged genes). **(G)** GSVA results show distinct pathways between the two clusters. **(H, I)** GO and KEGG annotation of DEGs.

We performed unsupervised clustering for the identification of the CIN-related clusters in the TCGA-BRCA cohort. When the K value of the cumulative distribution function was equal to 2, the consistency of the cluster analysis was the best. Therefore, we divided patients into 562 in cluster 1 and 529 in cluster 2 (Fig. 3A–C). Patients in cluster 1 expressed higher levels of CIN-related genes, as shown in the heatmap (Fig. 3D). Survival analysis suggested that the disease-free interval of cluster 2 was significantly prolonged than cluster 1 (*P* = 0.016; Fig. 3E).

According to the screening standard, 795 DEGs were identified (Fig. 3F). Then, the functional enrichment analysis was performed on the up-regulated DEGs in cluster 1. GSVA suggested that E2F targets, G2M checkpoints, and MYC targets were enriched in cluster 1 (Fig. 3G). Gene ontology (GO) analysis showed the enrichment of cell division, mitotic cell cycle, chromosome segregation, microtubule binding, and chromatin binding (Fig. 3H). The Kyoto Encyclopedia of Genes and Genomes (KEGG) annotation showed the significant enrichment of cell cycle, cytokine–cytokine receptor activation, and motor proteins (Fig. 3I). These findings implied that the DEGs mainly regulated the cell cycle, which may contribute to the differences in biological behavior and prognosis between cluster 1 and cluster 2.

### Construction and evaluation of the CIN score

**Figure 4:**
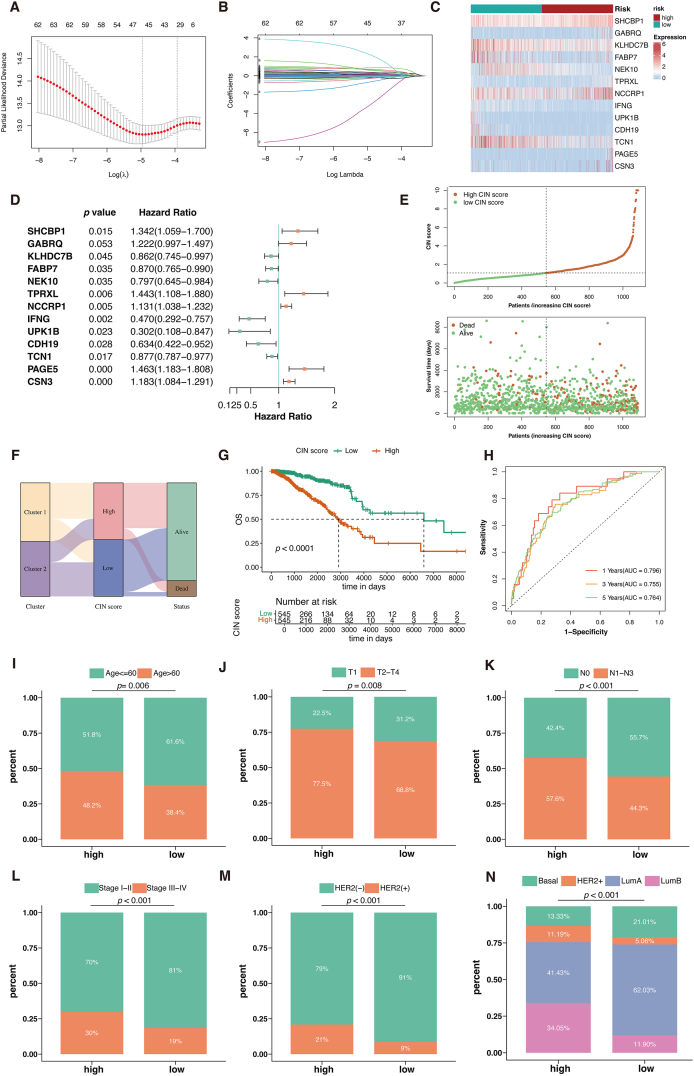
Construction of the CIN score. **(A, B)** Thirty-two genes were selected for multivariate COX regression analysis via the LASSO algorithm. **(C)** The expression of 13 genes in the two CIN score groups was visualized by a heatmap. **(D)** Forest plot of multivariate Cox results for 13 genes in the CIN score. **(E)** CIN score distribution is sorted from lowest to highest (upper). Survival status for each BC patient is categorized by CIN score (lower). **(F)** Sankey diagram correlating clusters, CIN score, and BC survival status. **(G)** The Kaplan–Meier curve comparing overall survival (OS) between low- and high-CIN score groups. **(H)** ROC curves depicting predictive performance of CIN score for 1, 3, and 5-year OS. **(I–N)** The comparisons of common clinicopathological factors: age (I), T stage (J), N stage (K), pathological stage (L), HER2 status (M), and PAM50 subtype (N) between the two groups.

The univariate Cox regression analysis identified 64 of the 795 DEGs as the preliminary prognostic genes, which was then reduced to 32 genes using the least absolute shrinkage and selection operator (LASSO) regression (Fig. 4A and B). Finally, a multivariate Cox regression analysis identified 13 independent prognostic genes to construct a novel gene signature called CIN score (Fig. 4C and D). CIN score = $\sum_{i=1}^{n}{\text{Coe}}_{i}*{\text{Exp}}_{i}$, where Coei refers to the coefficients of the genes, and Expi refers to the mRNA expression of 13 genes.

Based on the result of multivariate Cox regression analysis, we calculated CIN score as follows: CIN score = 0.294 × SHCBP1 + 0.200 × GABRQ – 0.149 × KLHDC7B – 0.139 × FABP7 – 0.227 × NEK10 + 0.367 × TPRXL +0.123 × NCCRP1 – 0.755 × IFNG – 1.196 × UPK1B – 0.456 × CDH19 – 0.131 × TCN1 + 0.380 × PAGE5 + 0.168 × CSN3.

Based on the CIN score’s median value, TCGA-BRCA patients were divided into low- and high-CIN score groups. We sorted all patients from lowest to highest CIN score and displayed the survival outcome of each patient (Fig. 4E). The Sankey diagram showed that patients with higher CIN scores corresponded to poorer prognosis (Fig. 4F), which was supported by Kaplan–Meier analysis showing that the low-CIN score group had a significantly superior OS (*P* < 0.0001; Fig. 4G). To assess the predictive accuracy of the CIN score, ROC curves were generated for OS at 1, 3, and 5 years (Fig. 4H). The area under the curve (AUC) was 0.796, 0.755, and 0.764, respectively, demonstrating the good performance of the CIN score in predicting OS.

We also compared the clinical pathological parameters of the two groups. Figure 4I–N showed that the low-CIN score group had favorable clinical characteristics, such as more pathological stage I–II (*P* < 0.001), T1 (*P* = 0.008), N0 (*P* < 0.001), and HER2-negative (*P* < 0.001) patients.

### Development and assessment of a CIN score-based nomogram

**Figure 5:**
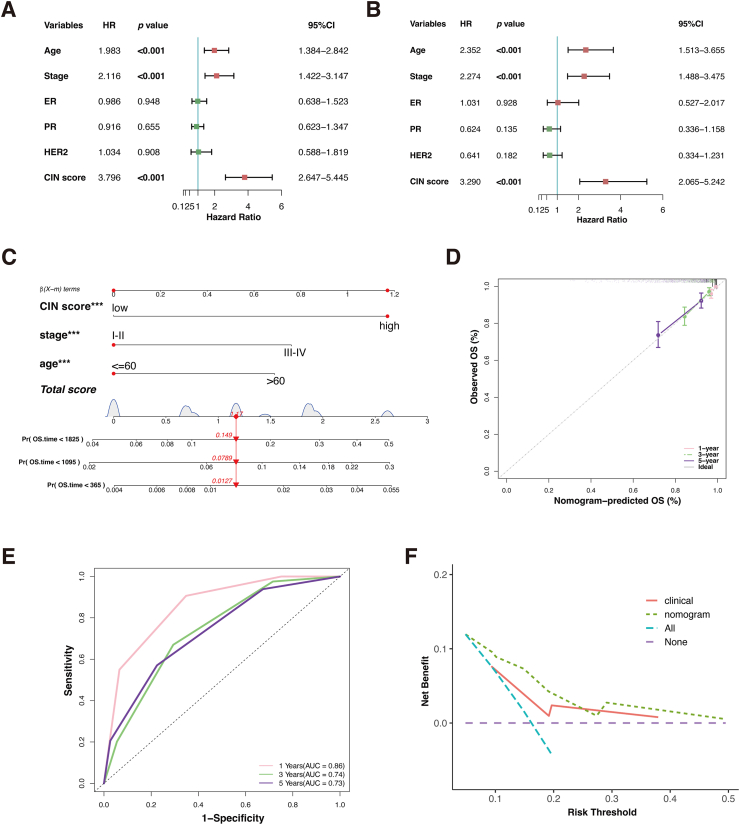
Construction and assessment of the nomogram. **(A, B)** Univariate and multivariate Cox regression analysis of the clinicopathological indicators and CIN score. **(C)** A comprehensive nomogram for predicting OS. **(D)** Calibration plots validate the stability of the model. **(E)** The ROC curves of the nomogram. **(F)** Decision curve analysis (DCA) of the clinicopathological indicators and the comprehensive nomogram. ∗*P* < 0.05, ∗∗*P* < 0.01, ∗∗∗*P* < 0.001, and ∗∗∗∗*P* < 0.0001.

Univariate and multivariate Cox regression analyses were performed to determine whether the CIN score could serve as an independent prognostic factor. The univariate Cox regression analysis showed that age (hazard ratio (HR): 1.983; 95% confidence interval (CI):1.384–2.842; *P* < 0.001), stage (HR: 2.116; 95% CI: 1.422–3.147; *P* < 0.001), and CIN score (HR: 3.796; 95% CI: 2.647–5.445; *P* < 0.001) were significantly correlated to BC prognosis (Fig. 5A). Multivariate Cox regression analysis revealed that age (HR: 2.352; 95% CI: 1.513–3.655, *P* < 0.001), stage (HR: 2.274; 95% CI: 1.488–3.475, *P* < 0.001), and CIN score (HR: 3.290; 95% CI: 2.065–5.242; *P* < 0.001) were independent predictors (Fig. 5B).

A comprehensive nomogram, which included the independent prognostic factors, was developed to predict the 1-, 3- and 5-year OS (Fig. 5C). The calibration curves demonstrated that the actual and predicted OS were highly consistent (Fig. 5D). The AUC values were 0.86, 0.74, and 0.73 at 1, 3, and 5 years, respectively (Fig. 5E). Finally, decision curve analysis examined the clinical benefit rate. Compared with the model with only clinical parameters, the clinical benefit rate of this comprehensive nomogram was higher, which could lead to improved clinical management (Fig. 5F).

### Functional enrichment analysis

**Figure 6:**
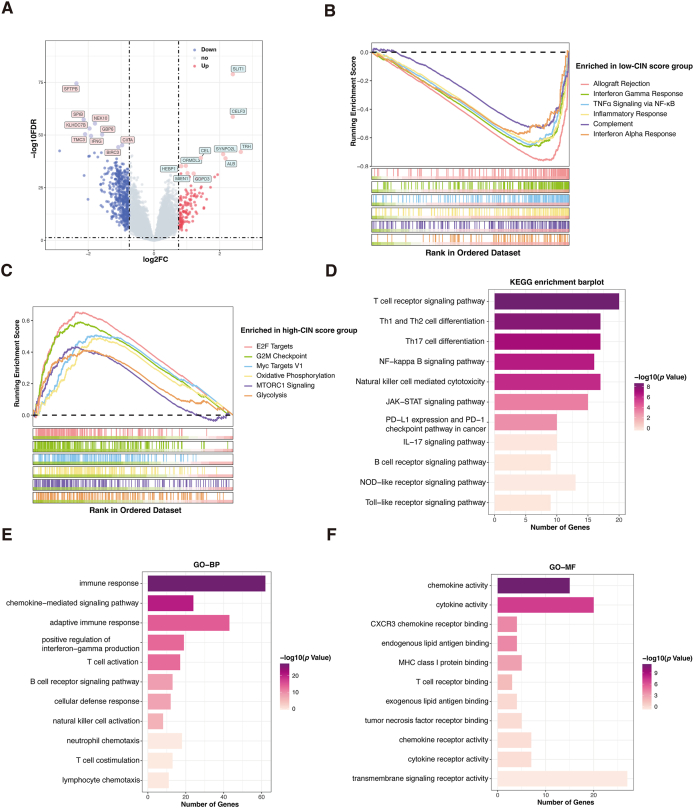
Differentially expressed genes (DEGs) and functional enrichment analysis. **(A)** Volcano plot showing the DEGs between the two groups. **(B, C)** The GSEA of the DEGs in the low-CIN score (B) and the high-CIN score (C) group. **(D)** KEGG enrichment analysis of down-regulated genes in the high-CIN score group. **(E)** GO analysis enriched down-regulated genes from biological processes in the high-CIN score group. **(F)** GO analysis enriched down-regulated genes from molecular functions in the high-CIN score group.

To determine the potential pathways that influence the prognosis of BC, we conducted DEGs analysis between the low- and high-CIN score groups. 625 DEGs were found, with 139 up-regulated and 486 down-regulated genes in the high-CIN score group (Fig. 6A). GSEA of DEGs revealed that the high-CIN score group was enriched in E2F targets, G2M checkpoint, and Myc targets. In contrast, the low-CIN score group was predominantly enriched in immune-related pathways, such as interferon-gamma response, TNF-α signaling via NF-κB, and inflammatory response (Fig. 6B and C). KEGG and GO analyses revealed that DEGs down-regulated in the high-CIN score group were enriched in immune-related pathways involved in the T cell signaling pathway, Th1 and Th2 cell differentiation, immune response, and chemokine activity (Fig. 6D–F). These functional analyses consistently implied that the tumor microenvironment in the high-CIN score group was immunosuppressed.

### Higher immune activity in the low-CIN score group

**Figure 7:**
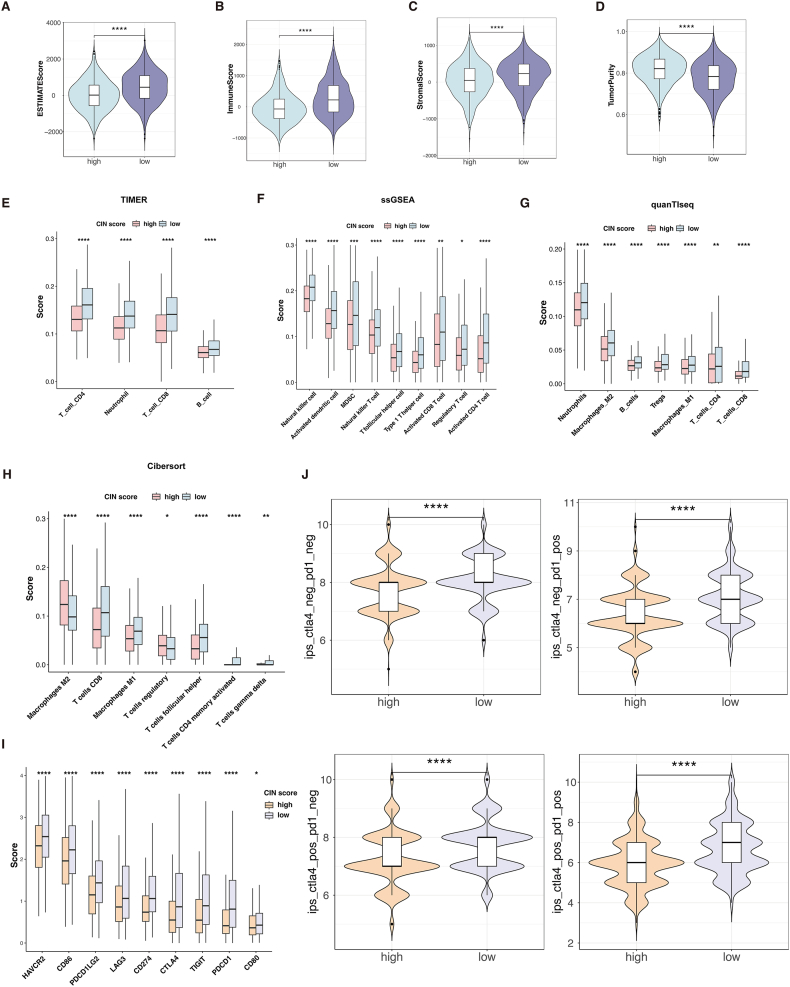
Immune infiltration and immune checkpoint gene expression across the two groups. **(A)** Comparison of the ESTIMATE score in the low- and high-CIN score groups. **(B)** Comparison of the immune score in the low- and high-CIN score groups. **(C)** Comparison of the stromal score in the low- and high-CIN score groups. **(D)** Comparison of the tumor purity in the low- and high-CIN score groups. **(E)** Comparisons of immunocyte infiltration fractions in the two groups using TIMER algorithms. **(F)** Comparisons of immunocyte infiltration fractions in the two groups using ssGSEA algorithms. **(G)** Comparisons of immunocyte infiltration fractions in the two groups using quanTIseq algorithms. **(H)** Comparisons of immunocyte infiltration fractions in the two groups using CIBERSORT algorithms. **(I)** The differential expression of checkpoint genes in the two groups. **(J)** Comparisons of the Immunophenoscore (IPS) in the two groups. ∗*P* < 0.05, ∗∗*P* < 0.01, ∗∗∗*P* < 0.001, and ∗∗∗∗*P* < 0.0001.

The enrichment analysis suggests that the immune landscape differs between the two groups. Therefore, we further explored the two groups’ immune cell abundance and immune function. ESTIMATE (Estimation of Stromal and Immune cells in Malignant Tumor tissues using Expression) data analysis revealed that the low-CIN score group had higher ESTIMATE scores (*P* < 0.05; Fig. 7A), immune scores (*P* < 0.05; Fig. 7B), stromal scores (*P* < 0.05; Fig. 7C), and lower tumor purity (*P* < 0.05; Fig. 7D), indicating a higher overall immune level and immunogenicity of the TIME in the low-CIN score group.

Utilizing four algorithms, “TIMER” (Fig. 7E), “ssGSEA” (Fig. 7F), “quanTIseq” (Fig. 7G), and “CIBERSORT” (Fig. 7H), we depicted variations in TIME components between the two groups. We found that immunosuppressive cells like regulatory T cells and M2 macrophage cells dominated the high-CIN score group. Conversely, the low-CIN score group exhibited enriched anti-tumor immune cell subpopulations, such as activated dendritic cells, activated CD4^+^ T cells, and CD8^+^ T cells, indicating that the low-CIN score group has an immune active microenvironment.

Furthermore, the low-CIN score group had significantly higher levels of nine immune checkpoint genes, including BTLA, PDCD1, and CTLA-4 (*P* < 0.05; Fig. 7I). Finally, the immunotherapy response in the two groups was compared using the IPS data, which showed the low-CIN score group had potentially better therapeutic benefits from ICI treatment (Fig. 7J).

### Drug sensitivity analysis

**Figure 8:**
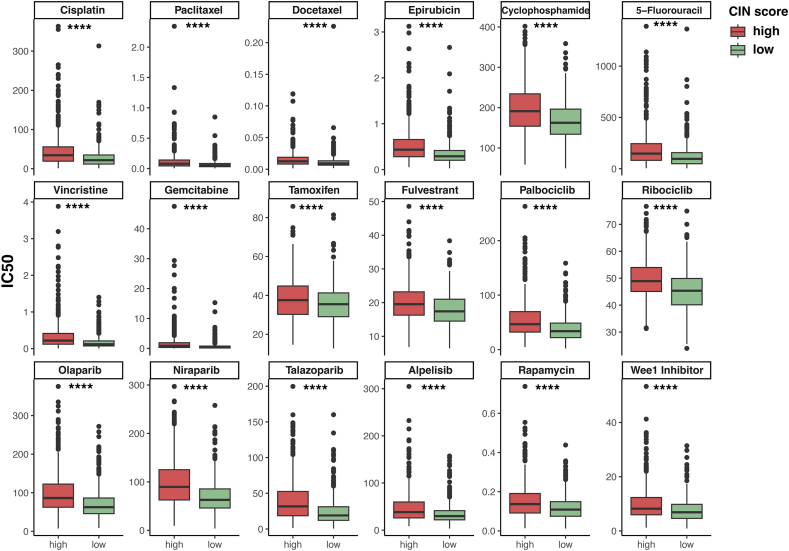
Drug sensitivity analysis. ∗*P* < 0.05, ∗∗*P* < 0.01, ∗∗∗*P* < 0.001, and ∗∗∗∗*P* < 0.0001.

The responsiveness of several commonly used chemotherapeutic, endocrine, and targeted agents was compared between the two groups to further investigate the clinical utility of the CIN score. We use the “oncoPredict” R package to calculate the IC_50_ values. The results showed that high-CIN score individuals were less responsive to all of the drugs, such as paclitaxel, docetaxel, cisplatin, cyclophosphamide, tamoxifen, palbociclib, and olaparib, compared with the low-CIN score counterparts (*P* < 0.05; Fig. 8). Therefore, some genes in CIN score may mediate drug resistance through some unknown mechanisms, which are worthy of experimental exploration.

### Validation of the CIN score in external databases

**Figure 9:**
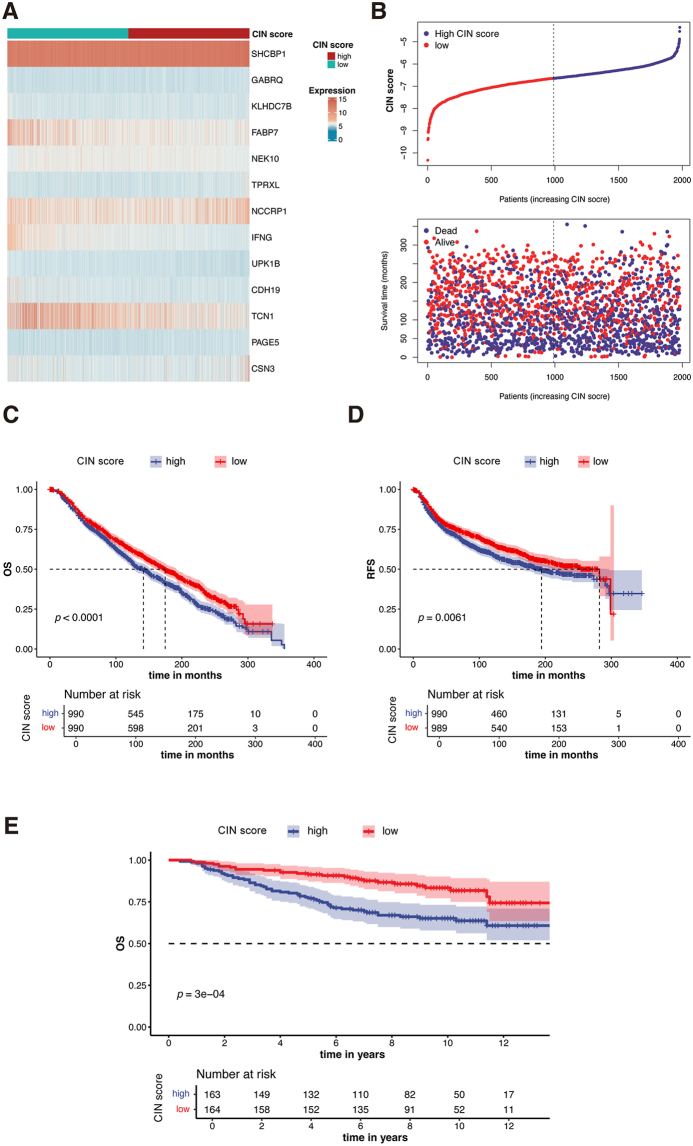
Validation of the CIN score in the METABRIC and GEO database. **(A)** Heatmap displaying expression levels of 13 genes in different CIN score groups. **(B)** Upper: CIN score distribution among BC patients, sorted from lowest to highest. Lower: Survival status is categorized by the CIN score for each BC patient. **(C, D)** Kaplan–Meier analysis comparing overall survival (C) and relapse-free survival (D) between high and low-CIN score groups in the METABRIC database. **(E)** Kaplan–Meier analysis comparing overall survival between high and low-CIN score groups in the GSE20658 dataset.

The METABRIC dataset was utilized to validate the prognostic value of the CIN score. The expression of 13 genes is displayed in Figure 9A. We calculated the CIN score for 1980 patients in this database, sorted them from lowest to highest, and displayed the survival status of each patient (Fig. 9B). Patients were separated into high- and low-CIN score groups based on the median of the CIN score. Kaplan–Meier survival analysis showed that patients in the high-CIN score group had significantly worse OS (*P* < 0.0001; Fig. 9C) and recurrence-free survival (*P* = 0.0061; Fig. 9D). Consistent with the findings in TCGA and METABRIC databases, CIN score was also correlated with worse prognosis of BC in GSE20658 (*P* = 0.0003; Fig. 9E).

### Expression pattern of the gene signature using scRNA-seq analysis

**Figure 10:**
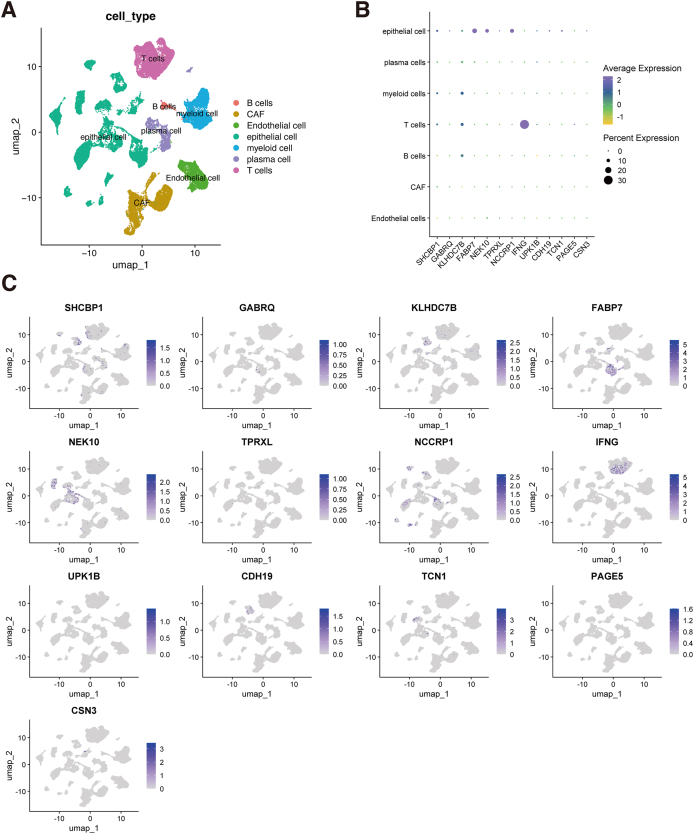
Expression pattern of the gene signature in the scRNA-seq dataset. **(A)** The UMAP analysis shows seven main cellular subtypes in the scRNA-seq data. **(B)** Dot plot of the expression of the gene signature in each cellular subtype. **(C)** The feature plot of the gene signature expression in each cellular subtype.

We analyzed a scRNA-seq dataset to determine whether the CIN score could be applied to distinguish between various cellular subtypes in BC. UMAP analysis identified seven cellular subtypes: B cells, cancer-associated fibroblasts, endothelial cells, epithelial cells, myeloid cells, plasma cells, and T cells (Fig. 10A). The gene signature expression in each cellular subtype was displayed in a dot plot (Fig. 10B) and a feature plot (Fig. 10C). These marker genes exhibited different expression patterns among the seven main cell types. IFNG was highly expressed in T cells. SHCBP1 was mainly expressed in epithelial, myeloid, and T cells. KLHDC7B was highly expressed in myeloid, T, and B cells. FABP7, NEK10, and NCCRP1 were specifically expressed in epithelial cells. Therefore, the genes in the CIN score exhibited relatively specific expression patterns and could be used to distinguish between various cellular subtypes.

**Figure 11:**
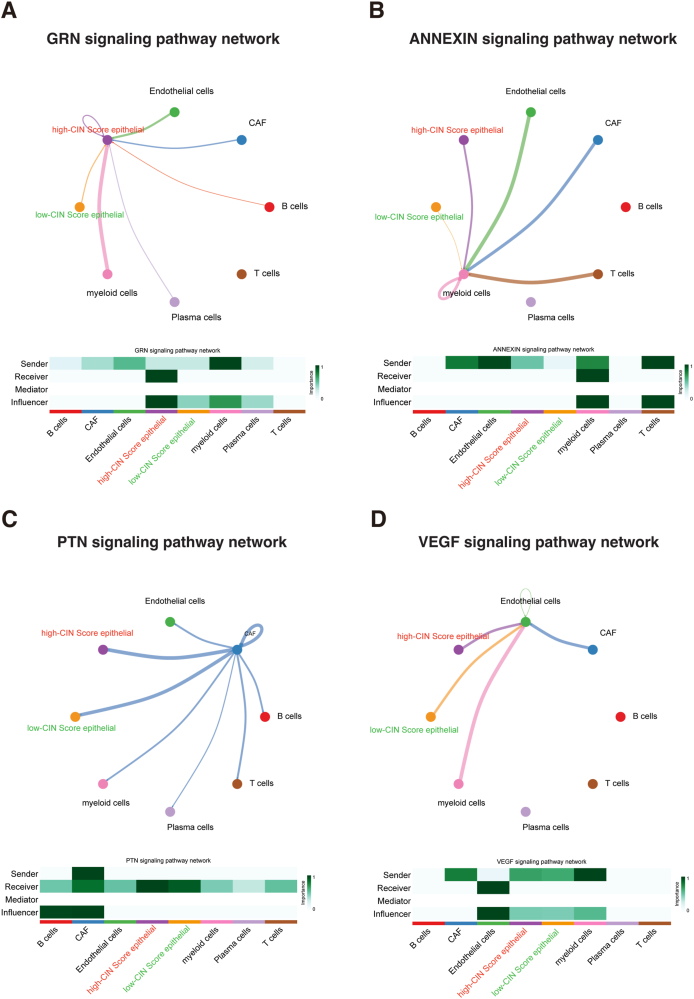
Cellular interaction analysis by CellChat. The cellular interaction network identified cell clusters in various signaling pathways, including **(A)** GRN, **(B)** ANNEXIN, **(C)** PTN, and **(D)** VEGF.

To determine the influence of CIN score on TIME, we divided epithelial cells into high- and low-CIN score clusters based on the CIN score expression. Subsequently, we calculated the cell–cell interactions among eight cell types. The relationship between CIN score expression and specific signaling pathways was further clarified. We found that epithelial cells with elevated CIN score expression exhibited strong interaction with stromal cells through the GRN, ANNEXIN, PTN, and VEGF signaling pathways (Fig. 11). These findings suggest that BC patients with high CIN score are prone to progression and metastasis via the stronger interaction with stromal cells.

## Discussion

Our study is the first to comprehensively analyze the CIN25 gene signature in BC patients, construct a novel gene signature called the CIN score, and further evaluate its performance for predicting biological characteristics, prognosis, TIME components, and drug sensitivity of BC. By combining the CIN score with clinical indicators, a prognostic nomogram was constructed for the accurate prediction of OS.

Our study first segregated TCGA-BRCA tumors into two clusters using the CIN25 gene signature. Significant differences in clinical characteristics, survival probability, and biological function were found between cluster 1 and cluster 2. These findings imply the important role CIN plays in the development and evolution of BC. Consistent with our results, CIN has been proposed as an important driver of tumor heterogeneity and has been proposed to be associated with cancer evolution,^37^, ^38^, ^39^ TIME components,^40^^,^^41^ and resistance to multiple therapeutics.^42^^,^^43^ Evidence suggests that CIN may induce epithelial-to-mesenchymal transition.^44^ Moreover, TCGA tumor samples analysis reveals that aneuploidy is associated with a lower expression level of the immune signature, thus promoting tumor immune evasion.^24^ One study analyzing the relationship between aneuploidies and therapeutic response revealed that 31 arm-level aneuploidies were associated with chemotherapeutic responsiveness.^37^ Another study has connected immunotherapy resistance to aneuploidy and chromosomal loss-of-heterozygosity events, which may reduce the expression of immune-associated genes.^24^^,^^45^ The importance of targeting CIN in BC is also becoming increasingly appreciated.

Several clinical trials targeting CIN are underway. ENMD-2076 is an orally bioavailable small-molecule inhibitor with antiproliferative activity via inhibition of Aurora kinase A. A phase II clinical trial (NCT01639248) showed that TNBC partially responded to the Aurora A inhibitor ENMD-2076.^46^ BAY1161909, BAY1217389, and S81694 are potent and highly selective inhibitors of TTK1 (MPS1), which is a serine–threonine kinase that functions as a core component of the spindle assembly checkpoint. Trials are underway to test the combination of paclitaxel with these TTK1 inhibitors in TNBC patients (NCT02138812, NCT02366949, NCT03411161).^14^ Since the recent development of sequencing technology and bioinformatics methods, our research has thoroughly investigated the role of CIN in BC using machine learning methods. Our results imply that it is promising to develop novel therapeutics targeting BC by exploiting CIN.

Using machine learning methods, we proposed a CIN-related 13-gene signature, the CIN score from DEGs between CIN–C1 and CIN–C2. Interestingly, the oncogenic roles of some genes in the CIN score have been reported. For example, some genes have proliferative effects on cancer cells. The nuclear translocation of SHCBP1 promotes bladder cancer progression^47^ and trastuzumab resistance in gastric cancer.^48^ KLHDC7B promotes the proliferation of both BC^49^ and bladder urothelial carcinoma.^50^ NEK10 directly phosphorylates p53 on Y327, and NEK10 loss increases cellular proliferation and sensitivity to DNA-damaging agents in BC.^51^ By up-regulating the expression of c-myc and Sox4, UPK1B promotes the occurrence and progression of several cancer types.^52^ Moreover, FABP7 is a polyunsaturated fatty acid-binding protein, an attractive metabolic target in glioblastoma, breast, and colon cancer.^53^, ^54^, ^55^ Additionally, some genes could serve as prognostic biomarkers in cancer. CDH19 is a prognostic biomarker for both bladder cancer and glioblastoma.^56^^,^^57^ TCN1 is associated with a worse prognosis and could predict the efficacy of neoadjuvant chemotherapy for colon cancer.^58^ Whether other genes in the CIN score play a role in cancer has not been reported. Therefore, further exploration should be conducted if these genes and related pathways are altered in BC samples and identified as therapeutic targets.

Our analyses further supported that a high CIN score could indicate a poor prognosis of BC, partly because a high CIN score was related to enriched oncogenic pathways, less abundant immune-related pathways, and higher IC50 to multiple therapeutics. Oncogenic pathways, such as E2F targets, MYC targets, and MTORC1 signaling, were enriched in the high-CIN score group. The TIME is a complex ecosystem primarily composed of immune cells and stromal cells, which contribute to promoting or suppressing anti-tumor immunity.^59^^,^^60^ The importance of TIME in BC’s development, evolution, and metastasis is well acknowledged, with the increasing agreement that deciphering its components and dynamics could lead to more potent immunotherapies.^61^^,^^62^ Our study showed that the low-CIN score group was abundant in immune-related pathways. Specifically, the low-CIN score group enriched the T cell receptor signaling pathway, Th1 and Th2 cell differentiation, and immune response. Therefore, we further performed immune infiltration analyses.

Immune infiltration analyses revealed that the low-CIN score group had a higher immune score and more abundant infiltration of CD4^+^ T cells, CD8^+^ T cells, and M1 macrophages compared with the high-CIN score group, which was in line with previous research demonstrating that high infiltration of anti-cancer immune cells indicated a good clinical outcome.^63^, ^64^, ^65^ ICIs achieve good effects in patients with immune-enriched tumors, as signified by high tumor-infiltrating lymphocytes and immune checkpoint gene expression.^66^, ^67^, ^68^ Not surprisingly, we also found that tumors in the low-CIN score group expressed higher levels of the immune checkpoint genes, such as CTLA-4, PD-1, and PD-L1, suggesting that the CIN score could help distinguish BC patients with different immunotherapy sensitivity. Collectively, we comprehensively illustrated that a higher CIN score represented a more aggressive phenotype, a more defective anti-tumor immunity, and less drug responsiveness of BC, which may contribute to the poor prognosis of the high-CIN score group.

Our finding that a high CIN score represents a suppressive TIME is supported by many reported studies. First, chromosomal missegregation and nuclear membrane rupture during mitosis can lead to the accumulation of cytosolic DNA, which activates the cGAS-STING pathway. Persistent cGAS-STING signaling results in the up-regulation of immune checkpoint molecules such as PD-L1, contributing to immune evasion and metastasis.^69^^,^^70^ Second, CIN can promote tumor heterogeneity and immunoediting, fostering the outgrowth of subclones with reduced immunogenicity that can evade immune surveillance.^71^ Third, to sustain continuous DNA damage repair under replication stress, CIN-high cells undergo metabolic reprogramming. This competition for essential metabolites (such as glucose and glutamine) creates a nutrient-depleted, metabolically hostile microenvironment that directly suppresses the effector functions of cytotoxic T cells.^72^^,^^73^ Finally, CIN-induced genomic instability may accelerate the acquisition of mutations in drug targets and resistance pathways, thereby limiting the efficacy of targeted therapies.^71^

The CIN25 signature is a well-established, pan-cancer biomarker for chromosomal instability. To develop a more precise tool tailored for BC, we refined this list using rigorous bioinformatic selection (including unsupervised consensus clustering and LASSO regression). The significant overlap (*e.g.*, UBE2C) confirms that our model retains the core biology of CIN. The unique genes in our signature likely represent BC-specific drivers or consequences of CIN. As a result, our optimized 13-gene CIN score demonstrates superior performance over the broader CIN25 signature in predicting BC-specific prognosis, immune infiltration, and drug resistance, providing more clinically actionable insights.

This study has some limitations. First, it was based on public databases, and further multicenter randomized controlled trials are needed to validate our results. Second, determining the biological function of certain CIN score genes requires both *in vitro* and *in vivo* experiments. Drug sensitivity analysis based on bioinformatics algorithms indicates that BC patients with a high CIN score are less sensitive to multiple therapeutics, which should be further validated by cell viability assays. Additionally, we employed multiple algorithms (TIMER, CIBERSORT, quanTIseq, and ssGSEA) to estimate immune cell infiltration. While there are several differences in the results due to methodological differences, the consistent directional trends of the main anti-tumor immune cells (CD4^+^ T cells and CD8^+^ T cells) observed across all methods between high- and low-CIN score groups strengthen the robustness of our conclusions regarding the immune microenvironment. The effect of CIN score on the infiltration of immunosuppressive cells, like regulatory T cells and M2 macrophage cells, should be further explored in the future.

In conclusion, our research comprehensively analyzed the CIN25 gene signature in BC and innovatively proposed the CIN score. CIN score performed well in predicting patient survival, TIME, and drug resistance. PCR method can measure the expression of 13 genes in the CIN score, making it both time- and cost-effective and easy to apply in clinical routine. Incorporating measures of the CIN score may help us better identify BC patients with a high risk of progression. Taken together, the present findings could shed light on more effective clinical strategies in the era of precision medicine of BC.

## CRediT authorship contribution statement

**Huiling Wang:** Formal analysis, Conceptualization. **Huijuan Dai:** Methodology. **Yaohui Wang:** Methodology. **Qiong Wu:** Validation. **Mingxi Zhu:** Validation. **Wenjin Yin:** Conceptualization. **Jinsong Lu:** Conceptualization.

## Data availability

The datasets analyzed in this study are available in GEO, METABRIC, and TCGA repositories, including GSE176078, METABRIC, and TCGA-BRCA.

## Funding

This study was funded by the 10.13039/501100001809National Natural Science Foundation of China (No. 82103695, 82303865, 82173115), 10.13039/501100013105Shanghai Rising-Star Program (No. 22QC1400200), Shanghai Municipal Health Commission Health Industry Clinical Research Special Project (China) (No. 202340085), and the Nurturing Fund of Renji Hospital (Shanghai, China) (No. PYIII20-09).

## Conflict of interests

The authors declared no conflict of interests.
